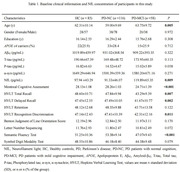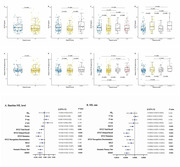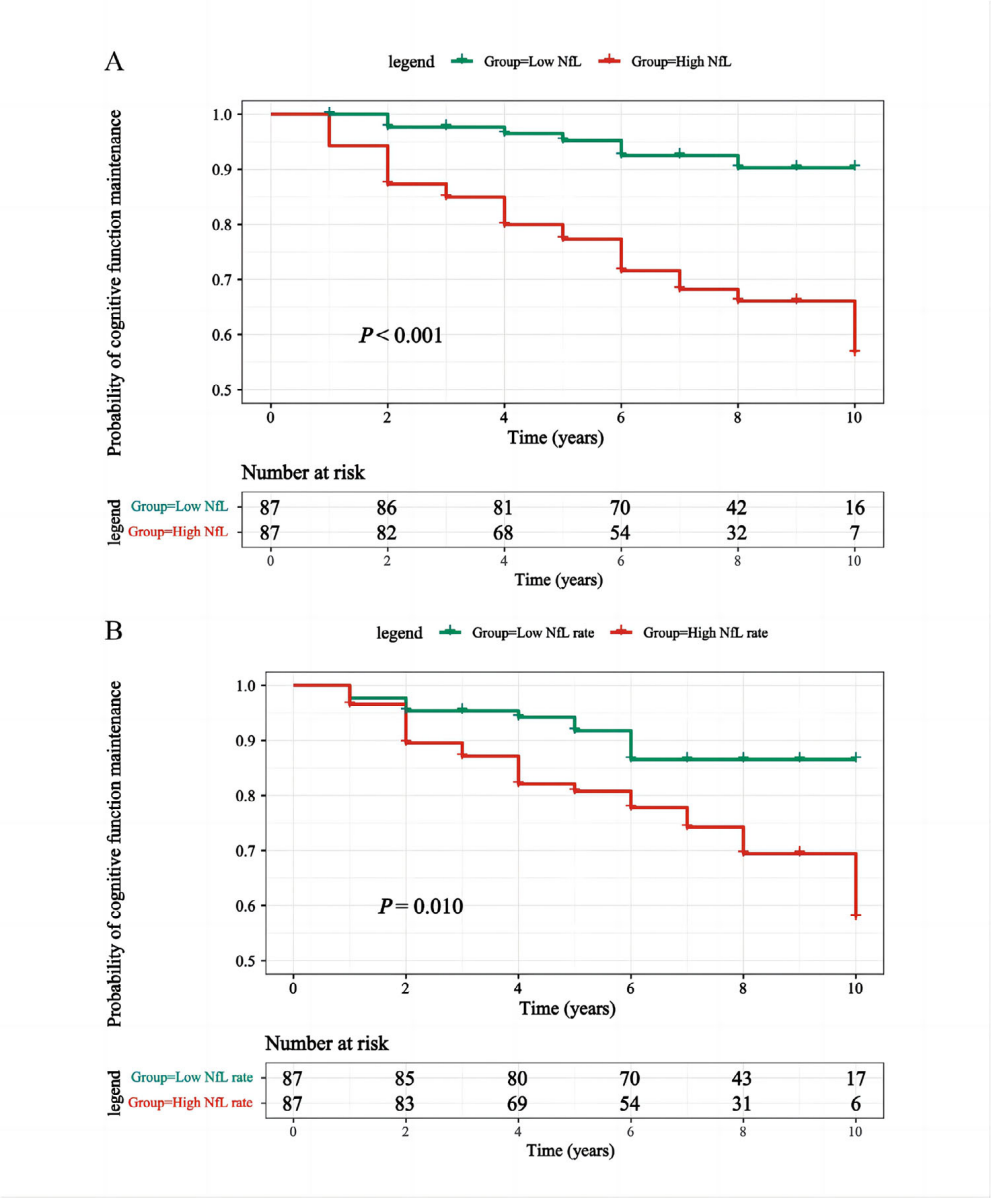# Cerebrospinal Fluid Neurofilament Dynamic Profiles Predict Cognitive Progression in Individuals with de novo Parkinson’s Disease

**DOI:** 10.1002/alz.088278

**Published:** 2025-01-09

**Authors:** Ze‐Hu Sheng, Lingzhi Ma, Fan Guo, Jia‐Yao Liu, Lan Tan

**Affiliations:** ^1^ Department of Geriatrics, The First Affiliated Hospital of Chongqing Medical University, Chongqing Medical University, Chongqing, Chongqing China; ^2^ Department of Neurology, Qingdao Municipal Hospital, Qingdao University, Qingdao, Shandong China; ^3^ Qingdao university, Qingdao China; ^4^ Department of Neurology, Qingdao Municipal Hospital, Qingdao university, Qingdao, Shandong China; ^5^ Qingdao Municipal Hospital, Qingdao China; ^6^ Qingdao Municipal hospital, Qingdao university, Qingdao, Shandong China

## Abstract

**Background:**

Neurofilament light chain protein (NfL) in cerebrospinal fluid (CSF) reflects the severity of neurodegeneration, with its altered concentrations discovered in Parkinson's disease (PD) and Parkinson's disease dementia (PD‐D).

**Method:**

A total of 259 people were recruited in this study, including 85 healthy controls (HC) and 174 neonatal PD patients from the Parkinson's Progression Markers Initiative (PPMI). Multiple linear regression and linear mixed effects models were used to examine the associations of baseline/ longitudinal CSF NfL with cognitive decline and other CSF biomarkers. Kaplan‐Meier analysis and log‐rank test were used to compare the cumulative probability risk of cognition progression during the follow‐up. Multivariate cox regression was used to detect cognitive progression in de novo PD.

**Result:**

We found PD patients with mild cognitive impairment (PD‐MCI) was higher than with normal cognition (PD‐NC) in terms of CSF NfL baseline levels (P = 0.003) and longitudinal increase rate (P = 0.034). Both baseline CSF NfL and its rate of change predicted measurable cognitive decline in de novo PD (MoCA, β = ‐0.010, P = 0.011; β = ‐0.0002, P < 0.001, respectively). The predictive effects in de novo PD patients aged > 65, male, ill‐educated (< 13 years) and without carrying Apolipoprotein E ε4 (APOE ε4) seemed to be more obvious and reflected in more domains investigated. We also observed that CSF NfL levels predicted progression in de novo PD patients with different cognitive diagnosis and amyloid status. After an average follow‐up of 6.66 ± 2.54 years, higher concentration above the median of baseline CSF NfL was associated with a future high risk of PD with dementia (adjusted HR 2.82, 95% CI: 1.11‐7.20, P = 0.030).

**Conclusion:**

Our results indicated that CSF NfL is a promising prognostic predictor of PD, and its concentration and dynamics can monitor the severity and progression of cognitive decline in de novo PD patients.